# A Mendelian randomization study of serum uric acid with the risk of venous thromboembolism

**DOI:** 10.1186/s13075-023-03115-6

**Published:** 2023-07-19

**Authors:** Lixian Ji, Peng Shu

**Affiliations:** 1 Department of Rheumatology, The Fourth Affiliated Hospital, Zhejiang University School of Medicine, Yiwu, Zhejiang 322000 China; 2 Department of Orthopedic Surgery, The Fourth Affiliated Hospital, Zhejiang University School of Medicine, Yiwu, Zhejiang 322000 China

**Keywords:** Uric acid, Venous thromboembolism, Mendelian randomization

## Abstract

**Background:**

Observational studies have linked hyperuricemia with venous thromboembolism (VTE). We aimed to investigate whether there are causal relationships between uric acid levels and VTE and its subtypes, including deep venous thrombosis (DVT) of the lower extremities and pulmonary embolism (PE).

**Methods:**

We utilized Mendelian randomization (MR) analysis to estimate the causal association in European individuals. We extracted two sets of polygenic instruments strongly associated (*p* < 5 × 10^−8^) with uric acid from the CKDGen consortium and UK biobank, respectively. Genetic associations with the risk of VTE, DVT, and PE were obtained from the FinnGen biobank. We used the inverse-variance weighted method as the preliminary estimate. Additionally, we employed MR-Egger, weighted median, and Mendelian randomization pleiotropy residual sum and outlier method as complementary assessments. Sensitivity analyses were performed to test for pleiotropic bias.

**Results:**

The genetically instrumented serum uric acid levels had no causal effects on VTE, DVT, and PE. Two sets of polygenic instruments used for exposure, along with three complementary MR methods, also yielded no significant association.

**Conclusions:**

Our MR analysis provided no compelling evidence for a causal relationship of serum uric acid with the risk of VTE. This suggests that uric acid-lowering therapies in patients with hyperuricemia may not be effective in reducing the likelihood of developing VTE.

**Supplementary Information:**

The online version contains supplementary material available at 10.1186/s13075-023-03115-6.

## Introduction

Venous thromboembolism (VTE) is a chronic condition that manifests as either deep vein thrombosis (DVT) or pulmonary embolism (PE), depending on the location of the clot [1]. With an annual incidence rate of 1–2 cases per 1000 population [2, 3], it is a significant cause of morbidity and mortality worldwide, second only to ischemic heart disease and stroke in its contribution to the global burden of cardiovascular disease [4]. The development of VTE involves multiple factors, including inherited genetic factors and acquired factors such as aging, surgery, and prolonged immobilization [5, 6].

Uric acid (UA), the end product of purine breakdown, has been shown to modulate the interplay between compromised endothelial function, inflammatory response, and thrombogenicity [7]. Most observational and experimental studies have indicated that hyperuricemia increases the risk of VTE. However, these associations may be confounded by multiple factors that are difficult to adequately consider in these studies, and the risk of reverse causality cannot be avoided.

Mendelian randomization (MR) is a valuable statistical technique that employs genetic variants as instrumental variables (IVs) to establish causal relationships between risk factors (exposure) and diseases (outcome). MR is particularly useful when randomized controlled trials (RCTs) are infeasible or unethical [8]. In the case of serum UA level, a complex trait influenced by genetic factors, twin and family studies estimate its heritability at 40–60% [9, 10]. Genetic variants as instruments for serum UA level or gout enable causal inference in many MR studies. In a previous two-sample MR study, the causal links of several blood metabolites, including UA, on the risk of VTE were investigated [11]. However, they did not account for population stratification in their study design as the exposure of UA they used was distinct from the outcome variable based on race. In this study, we conducted a comprehensive MR analysis to infer the causality of hyperuricemia on VTE based on exposure (serum UA) and three outcomes (VTE, DVT, and PE).

## Methods

### Study design

**Fig. 1:**
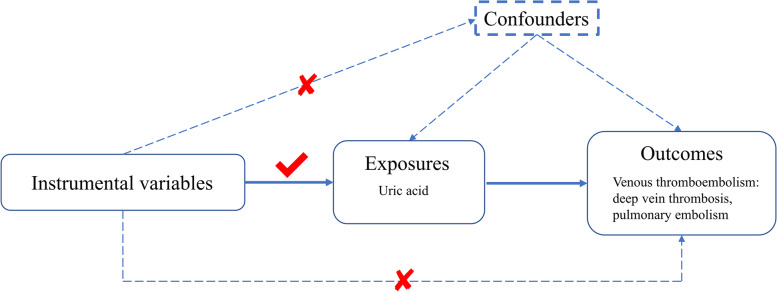
An overview of the study design

MR utilizes single-nucleotide polymorphisms (SNPs) as IVs, which should satisfy the three core assumptions [8]. First, IVs must exhibit strong associations with exposure. Second, genetic variants must be independent from unmeasured confounding factors that may affect the exposure-outcome association. Lastly, IVs are presumed to affect the outcome only through their associations with exposure, thus minimizing the potential for pleiotropic effects (Fig. 1).

### Data sources

Summary data for UA were obtained from the Chronic Kidney Disease Genetics (CKDGen) consortium, which conducted the most recent genome-wide association study (GWAS) of urate levels in 288,649 European participants [12]. To validate the robustness of the results and minimize bias, an additional dataset of urate levels from 343,836 participants was selected from the UK biobank (UKB), which can be downloaded from https://gwas.mrcieu.ac.uk/datasets (ID: ukb-d-30880_irnt).

Summary statistics for VTE, DVT of the lower extremities, and PE in European populations were obtained from the FinnGen consortium (Release 8, https://r8.finngen.fi/) [13]. Case identification was established based on International Classification of Diseases codes.

### Instrumental variable selection

**Fig. 2:**
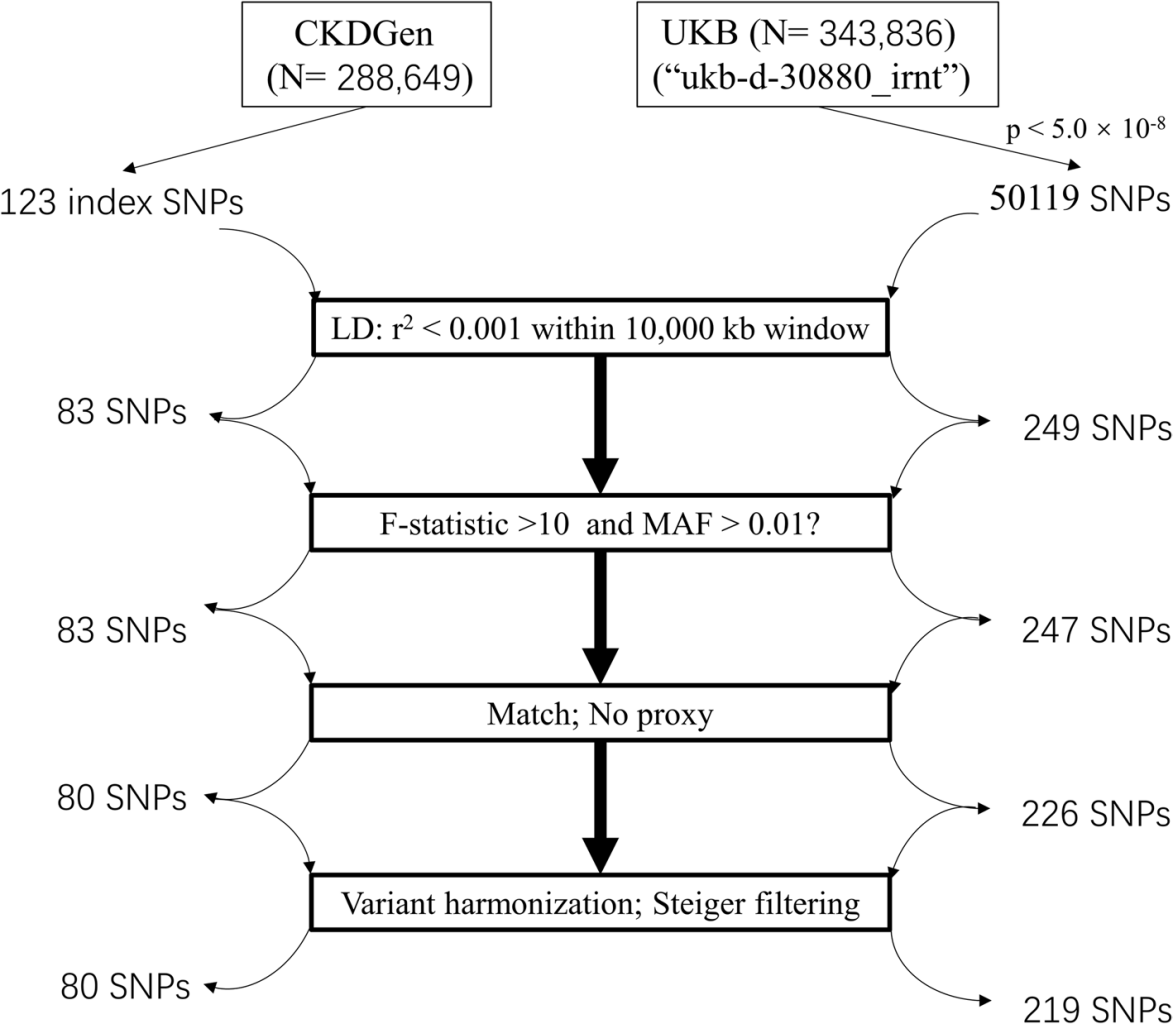
The flowchart of instrumental variable selection. CKDGen, the Chronic Kidney Disease Genetics consortium; UKB, UK biobank. SNPs, single-nucleotide polymorphisms. LD, linkage disequilibrium. MAF, minor allele frequency

Initially, genome-wide significant SNPs for UA were identified with a threshold of *p* < 5.0 × 10^−8^. Subsequently, a set of predefined parameters (*r*^2^ < 0.001 within 10,000 kb window; European 1000 Genomes Panel) was utilized to exclude SNPs in linkage disequilibrium, ensuring the independence of the selected IVs. The strength of the IVs was assessed using the *F*-statistic, where values greater than 10 were indicative of a reduced likelihood of weak instrument bias [14]. Finally, SNPs that could not be matched to the outcome datasets and palindromic SNPs were removed during the variant harmonization process. In addition, we used the MR Steiger filtering to remove SNPs with an incorrect causal direction [15]. Palindromic SNPs are defined as the A/T or G/C allele and minor allele frequency between 0.01 and 0.30. The flowchart of instrumental variable selection is shown in Fig. 2.

### Statistical analysis

The main analysis utilized the multiplicative random-effects inverse-variance weighted (IVW) method, which combines cumulative causal estimates of Wald ratios derived from each IV [8]. Supplementary analyses were performed using MR-Egger, weighted median, and the Mendelian randomization pleiotropy residual sum and outlier (MR-PRESSO) method to ensure the validity and robustness of the findings, given the potential for invalid instrument bias or pleiotropy to impact the IVW estimates. The weighted median method provides consistent estimates even when up to 50% of the weight is derived from invalid SNPs [16]. The MR-Egger method provides reliable results even when all SNPs are invalid, but it is less effective compared to the IVW method [17]. Additionally, the MR-PRESSO method can detect pleiotropy and provide corrected causal inferences [18].

Pleiotropy was assessed through the utilization of the MR–Egger intercept test and MR-PRESSO global test. MR–Egger intercept test examines the presence of a non-zero intercept to assess the average pleiotropic effect of IVs [17]. To evaluate potential heterogeneity, the Cochran’s *Q* test in the IVW approach was employed. The leave-one-out analysis involved systematically removing one SNP at a time was used to evaluate the influence of each individual variant on the outcomes and to detect any potential outliers.

The statistical analyses were conducted using the R software (version 4.0.2) and the TwoSampleMR (version 0.5.6) and MR-PRESSO packages. A Bonferroni-corrected significance threshold of *p* < 0.017 (0.05/3) was applied to determine statistical significance, with associations displaying a *p* value between 0.017 and 0.05 deemed suggestive evidence.

## Results

### Genetic instrumental variable selection

Based on a genome-wide significant threshold of *p* < 5 × 10^−8^, a total of 50,242 SNPs for UA (123 index SNPs from the CKDGen consortium and 50,119 SNPs from UKB) were identified. After adjusting for linkage disequilibrium, we obtained 332 independent SNPs for UA (83 from the CKDGen consortium and 249 from the UKB). Subsequently, rigorous screening was conducted as described earlier, leading to the identification of 80 SNPs (total *R*^2^ of 4.9%) for the associations between UA-CKDGen with VTE, DVT, and PE; 219 SNPs (total *R*^2^ of 7.7%) for the associations between UA-UKB with VTE, DVT, and PE, respectively. All *F*-statistic values for the obtained IVs were greater than 10, indicating no significant weak IV bias. The information of these genetic variants utilized in the MR analyses is detailed in Supplementary Table 1.

### Effects of genetically proxied serum urate on VTE, DVT and PE

**Fig. 3:**
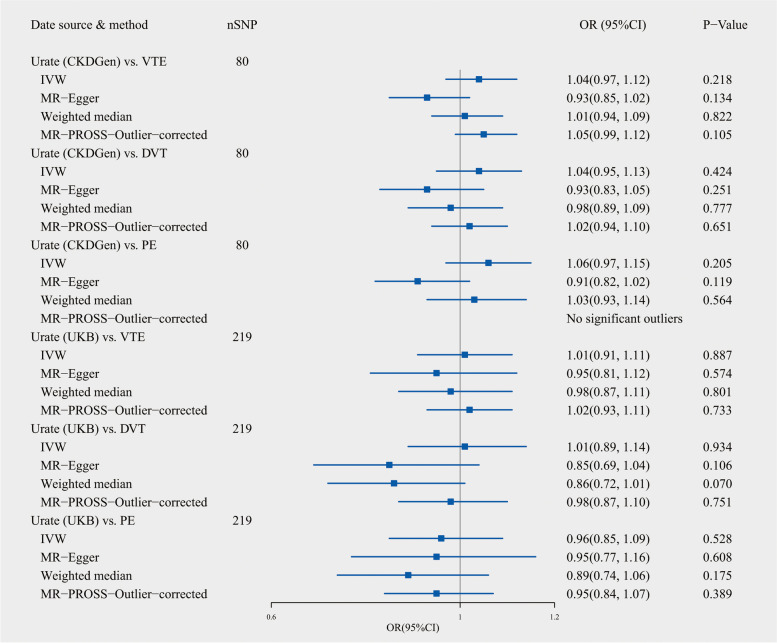
Estimates for the causal effect of genetically predicted uric acid levels on venous thromboembolism. CKDGen, the Chronic Kidney Disease Genetics consortium; UKB, UK biobank; VTE, Venous thromboembolism; DVT, deep vein thrombosis; PE, pulmonary embolism. IVW, inverse-variance weighted method

Figure 3 displays the causal estimate of UA on VTE, DVT, and PE. Using genetic variants from the CKDGen consortium, the random-effects IVW method showed that genetically proxied serum UA had a null association with the risk of VTE [odds ratio (OR): 1.04; 95% confidence interval (CI)), 0.97~1.12; *p* = 0.218], DVT (OR:1.04; 95% CI, 0.95~1.13; *p* = 0.424), and PE (OR:1.06; 95% CI, 0.97~1.15; *p* = 0.205). Despite significant pleiotropy and heterogeneity being identified by MR-Egger regression and Cochran’s *Q* test (Table 1), all MR analysis methods do not support significant causation. Re-administration of MR analysis using IVs from UKB produces similar results. Moreover, the results remain unchanged after removal of MR-PROSS-identified outliers. The leave-one-out tests did not indicate a potential causality after excluding any one SNP (Supplementary Figures 1-6).

**Table 1:** Pleiotropy and heterogeneity tests for the causal link between serum urate levels and the risk of venous thromboembolism

	Pleiotropy			Heterogeneity
	MR-Egger intercept test		MR-PRESSO global test	Cochran’s *Q* test
				IVW
	Intercept	Pval	Pval	Pval
Urate (CKDGen) vs. VTE	0.009	9.88E−04	< 5e−04	1.44E−06
Urate (CKDGen) vs. DVT	0.008	0.017	0.0175	0.013
Urate (CKDGen) vs. PE	0.012	6.55E−04	0.014	0.013
Urate (UKB) vs. VTE	0.002	0.418	< 5e−04	4.28E−14
Urate (UKB) vs. DVT	0.006	0.038	< 5e−04	1.18E−05
Urate (UKB) vs. PE	4.46E−04	0.866	< 5e−04	6.20E−06

*CKDGen* The Chronic Kidney Disease Genetics consortium, *UKB* UK biobank, *IVW* Inverse variance weighted analysis, *VTE* venous thromboembolism, *DVT* Deep vein thrombosis of the lower extremities, *PE* Pulmonary embolism

## Discussion

We employed an MR design to elucidate the causal associations between serum UA concentration and the risk of VTE, including its subtypes DVT and PE. Our study results demonstrate that genetically determined UA has no causal link with VTE, DVT, or PE. These effects were consistent across the two sets of polygenic instruments used for exposure. These findings provide genetic evidence that hyperuricemia may not be predictive risk factors for VTE and emphasize the importance of high-quality, well-designed RCTs to further investigate this topic.

Most clinical observational studies support a positive association of UA with VTE risk [19–23]. However, one case-control study found a correlation between serum UA and VTE only among individuals with high levels of high-density lipoprotein cholesterol [24]. Despite these findings, it is important to note that these observational studies cannot establish a definitive causal link due to inherent limitations such as methodological shortcomings, small sample sizes, selection bias, and insufficient adjustment for confounders.

MR is a robust approach in human genetics research that enables causal inference between an exposure and a complex disease outcome. By utilizing genetic variants as IVs, MR can control for non-heritable environmental confounders and mitigate reverse causation bias. Our study aimed to investigate the causal relationships between UA and venous thrombosis using a two-sample MR design. Our analysis did not reveal a significant causal association between UA and venous thrombosis. Notably, our multiple MR models provided consistent results. We also followed established MR guidelines to mitigate bias, including selecting genetic variants from a different dataset as supplementary sources of IVs for exposure and applying Steiger filtering to remove instruments with reverse causal effects. Our additional re-MR analysis using these approaches confirmed the null conclusions. These findings indicate that our results are robust and strengthen the conclusions drawn from our MR study, despite discordance with most observational studies.

MR assumes that an IV affects the outcome only through the exposure, a condition known as the absence of horizontal pleiotropy. Horizontal pleiotropy occurs when a genetic variant influences other trait or the outcome directly, potentially biasing MR tests and leading to false causal relationships. In our study using the IVs from the CKDGen, a sensitivity analysis using the MR-Egger intercept test detected significant levels of pleiotropy. Further screening of IVs using the PhenoScanner database, adjusting for potential VTE risk factors such as body mass index, smoking, blood pressure, and blood lipids, consistently detected positive results for pleiotropic tests (Supplementary Tables 2-4). When pleiotropy was taken into account, our MR study yielded null causal inferences, in contrast to most previous prospective studies. In fact, the presence of pleiotropy typically results in non-null estimates, making a null finding more convincing [25]. Additionally, heterogeneity was minimized in our study by using multiplicative random-effects IVW as the main method of analysis.

According to published MR studies, it is noteworthy that most investigations do not provide evidence for a causal relationship between serum UA levels and cardiometabolic diseases, such as coronary heart disease, ischemic stroke, hypertension, heart failure, and type-2 diabetes [26–30], as well as other health outcomes including chronic kidney disease, Parkinson’s disease, bone mineral density, and cancer [31–35]. Our study also examined the VTE trait as the outcome variable and found no causal relationship. These findings suggest that serum UA may not induce an adverse cardiovascular disease phenotype, but rather a secondary phenomenon of it. The observed causation in observational studies may be influenced by residual confounding factors. Li et al. proposed a unique perspective to account for the discrepancy between MR studies and observational findings on the causality of UA on multiple diseases. They suggested that previous MR studies were generally driven by hypotheses and that the pleiotropy of genetic variants may contribute to the observed associations [28]. Nonetheless, there is an urgent need for rigorously designed research to further investigate these findings.

One of the major strengths of our study is the use of a large sample of GWAS dataset with an MR design to establish the causal relationship between UA and VTE. Moreover, we screened the IVs of exposure separately from two large GWAS datasets to minimize bias. However, our MR study also encounters some challenges that require attention. Firstly, our study cannot rule out the impact of pleiotropy. However, pleiotropy often causes the cause and effect to move in a non-null direction. Nevertheless, multiple MR statistical models considering pleiotropy produced consistent results in both of our two sets of polygenic analyses. Secondly, while the exposure and outcome data used in our study were obtained independently, there is a possibility of some sample overlap, which could inflate the type 1 error rate [36]. Nonetheless, the *F*-statistics of the IVs used were sufficiently large to mitigate the potential impact of weak instrumental bias. Thirdly, our study did not assess other nonlinear effects of UA, such as U-shaped effects [37], as the MR design assumed a linear correlation. Lastly, the GWAS data we used in this study were from the European population, thus our results may not be generalizable to other ethnic populations.

## Conclusion

In summary, our MR study found no causal effect of UA on VTE (including DVT and PE), suggesting the associations between UA and VTE observed in epidemiological studies may not indicate causality.

## Supplementary Information

**Additional file 1:** **Supplementary Table 1.** Genetic variants used in the MR analyses. **Supplementary Table 2.** SNPs associated with VTE risk factors (PhenoScanner V2 database, *p*<1E-05). **Supplementary Table 3.** MR Analysis after adjusting for potential VTE risk factors. **Supplementary Table 4.** Pleiotropy tests of urate instrumental variables after adjusting for potential VTE risk factors in GWAS for VTE and its subtypes.

**Additional file 2:** **Supplementary Figure 1.** The plots of “leave-one-out” analysis method to show the influence of individual SNP on the causal effect of genetically predicted uric acid (CKDGen consortium) on venous thromboembolism. **Supplementary Figure 2.** The plots of “leave-one-out” analysis method to show the influence of individual SNP on the causal effect of genetically predicted uric acid (UKB) on venous thromboembolism. **Supplementary Figure 3.** The plots of “leave-one-out” analysis method to show the influence of individual SNP on the causal effect of genetically predicted uric acid (CKDGen consortium) on deep venous thrombosis. **Supplementary Figure 4.** The plots of “leave-one-out” analysis method to show the influence of individual SNP on the causal effect of genetically predicted uric acid (UKB) on deep venous thrombosis. **Supplementary Figure 5.** The plots of “leave-one-out” analysis method to show the influence of individual SNP on the causal effect of genetically predicted uric acid (CKDGen consortium) on pulmonary embolism. **Supplementary Figure 6.** The plots of “leave-one-out” analysis method to show the influence of individual SNP on the causal effect of genetically predicted uric acid (UKB) on pulmonary embolism.

## Data Availability

The original contributions presented in the study are included in the article/supplementary material, further inquiries can be directed to the corresponding author.

## Abbreviations

VTE: Venous thromboembolism
DVT: Deep venous thrombosis
PE: Pulmonary embolism
CKDGen: The Chronic Kidney Disease Genetics consortium
UKB: UK biobank
UA: Uric acid
MR: Mendelian randomization
IVs: Instrumental variables
RCTs: Randomized controlled trials
SNPs: Single-nucleotide polymorphisms
GWAS: Genome-wide association study
IVW: Inverse-variance weighted method
MR-PRESSO: Mendelian randomization pleiotropy residual sum and outlier method
OR: Odds ratio
CI: Confidence interval
