# Supplementary material for: A Mendelian randomization study of serum uric acid with the risk of venous thromboembolism
Source: Arthritis Res Ther. 2023 Jul 19;25:122. doi: 10.1186/s13075-023-03115-6 (PMC10354911; doi:10.1186/s13075-023-03115-6)

**
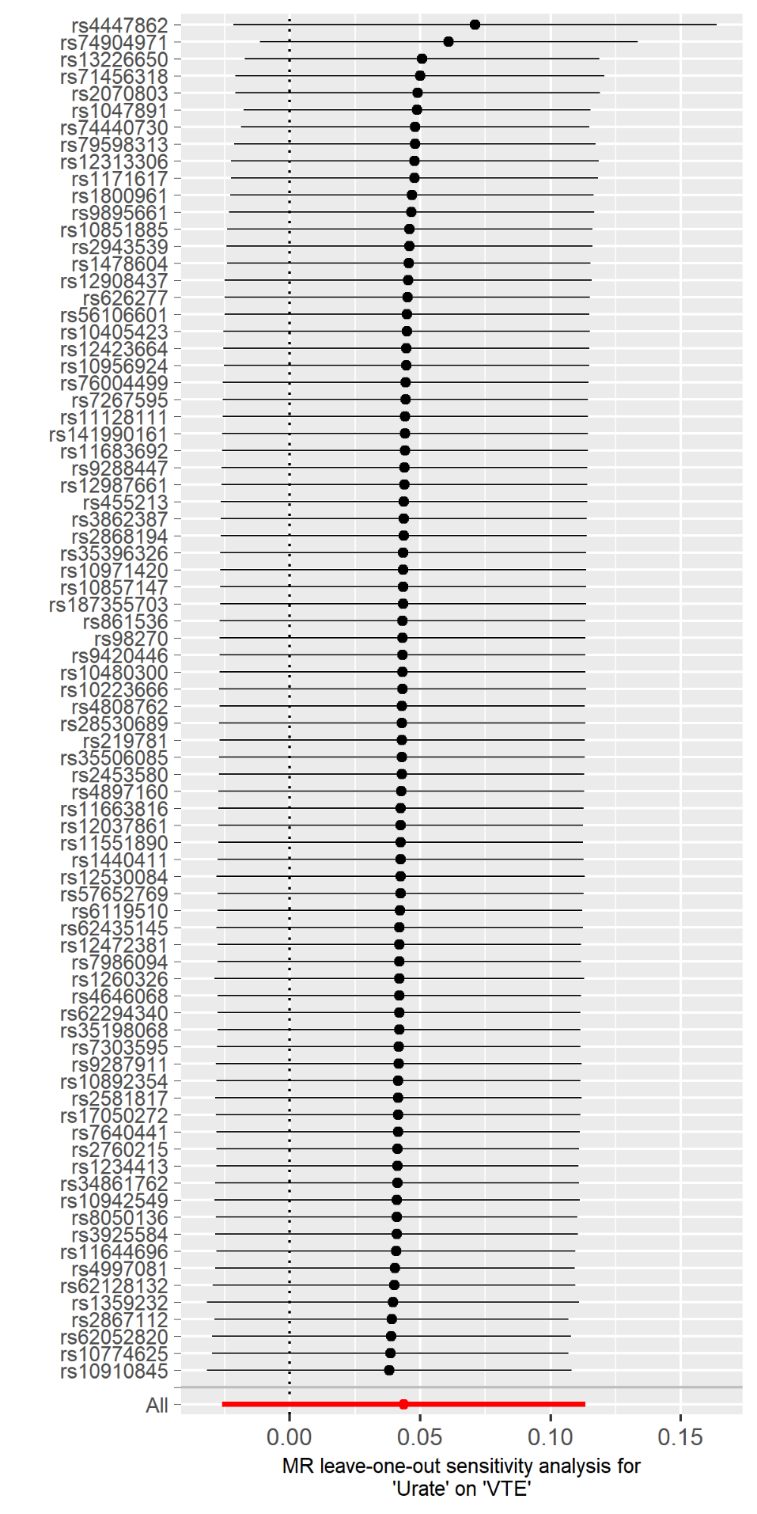
Supplementary Figure 1**. The plots of “leave-one-out” analysis method to show the influence of individual SNP on the causal effect of genetically predicted uric acid (CKDGen consortium) on venous thromboembolism.

**Supplementary Figure 2.** The plots of “leave-one-out” analysis method to show the influence of individual SNP on the causal effect of genetically predicted uric acid (UKB) on venous thromboembolism.


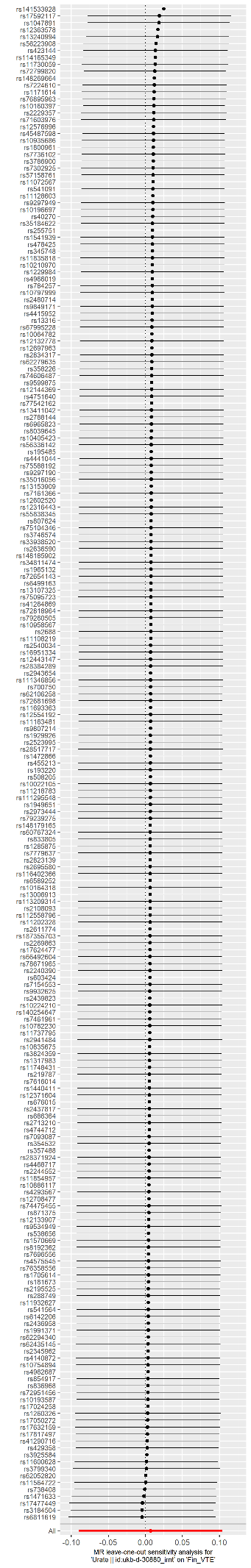


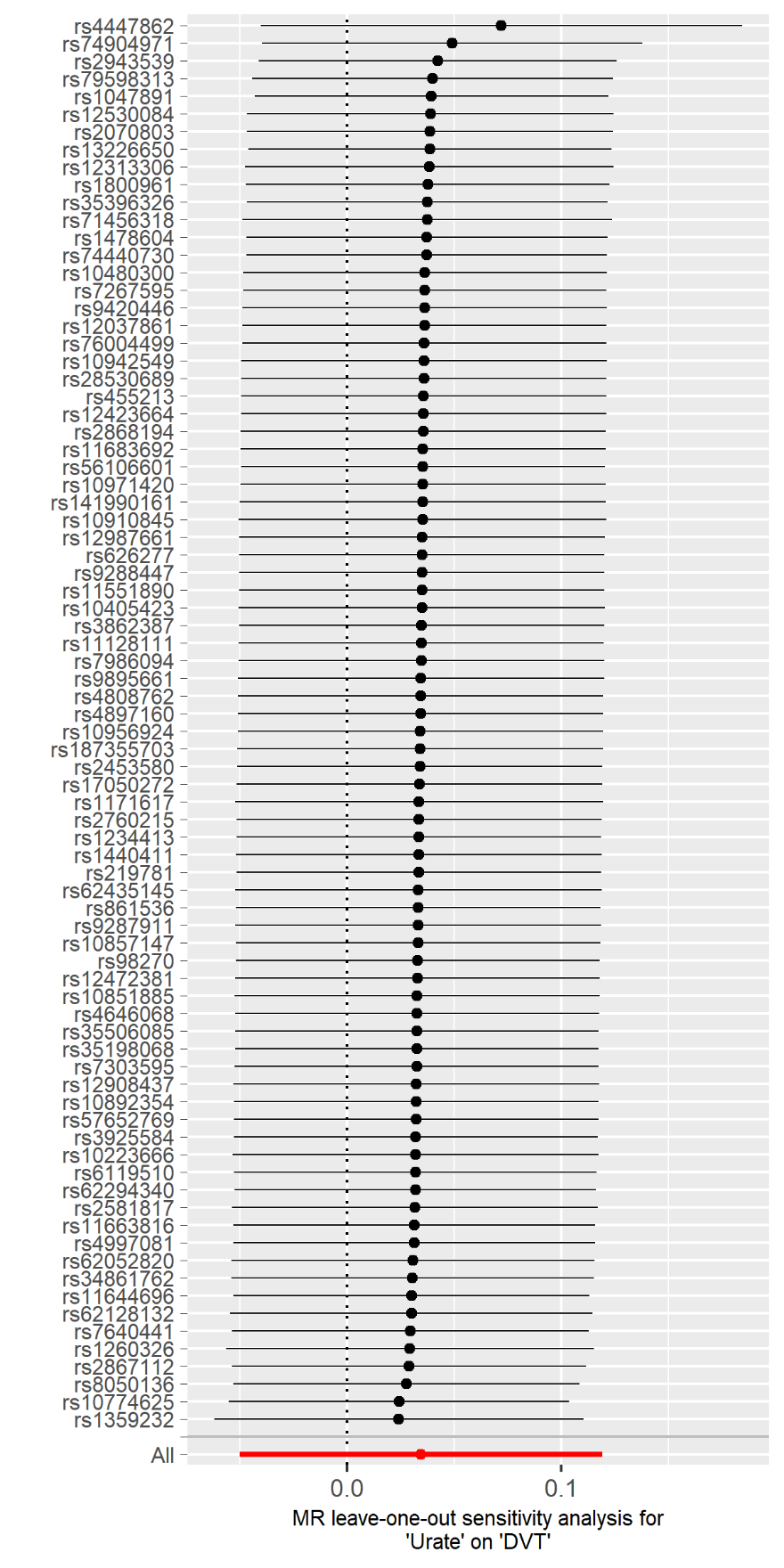
**Supplementary Figure 3.** The plots of “leave-one-out” analysis method to show the influence of individual SNP on the causal effect of genetically predicted uric acid (CKDGen consortium) on deep venous thrombosis.

**Supplementary Figure 4.** The plots of “leave-one-out” analysis method to show the influence of individual SNP on the causal effect of genetically predicted uric acid (UKB) on deep venous thrombosis.


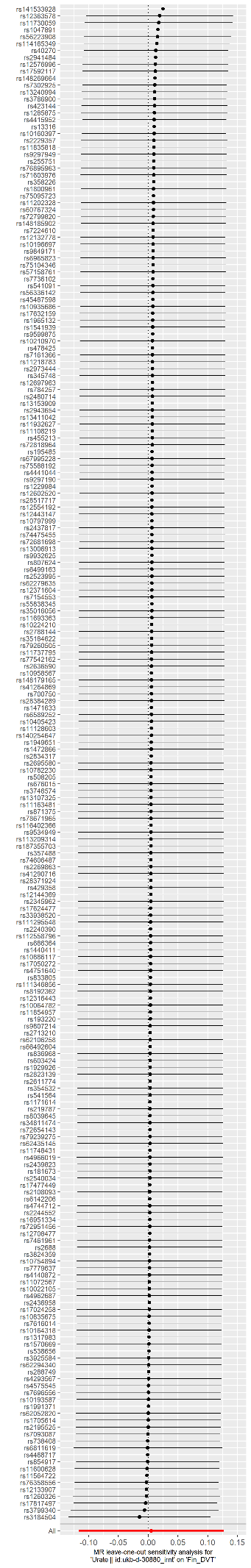


**Supplementary Figure 5.** The plots of “leave-one-out” analysis method to show the influence of individual SNP on the causal effect of genetically predicted uric acid (CKDGen consortium) on pulmonary embolism.


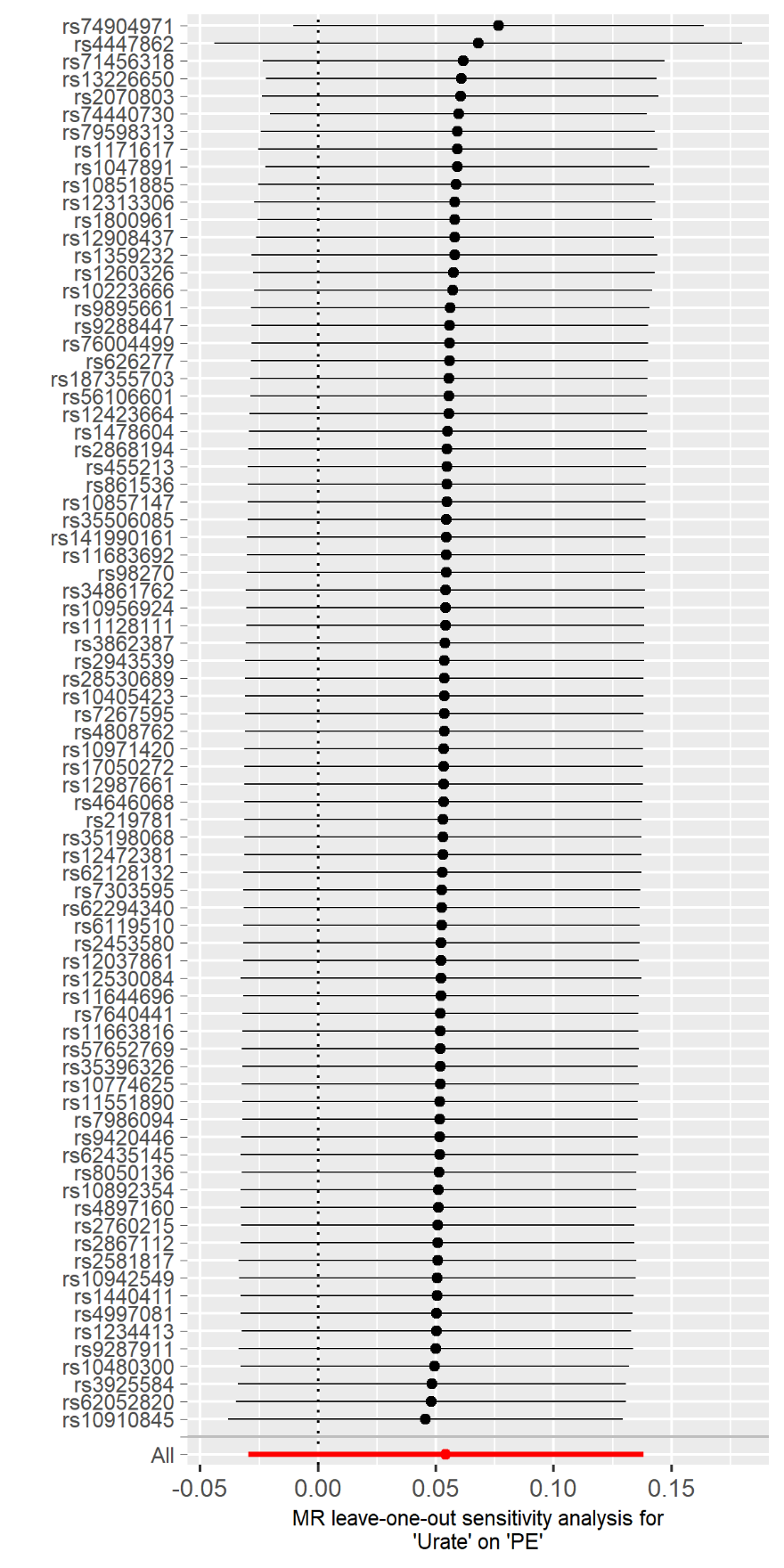


**Supplementary Figure 6**. The plots of “leave-one-out” analysis method to show the influence of individual SNP on the causal effect of genetically predicted uric acid (UKB) on pulmonary embolism.


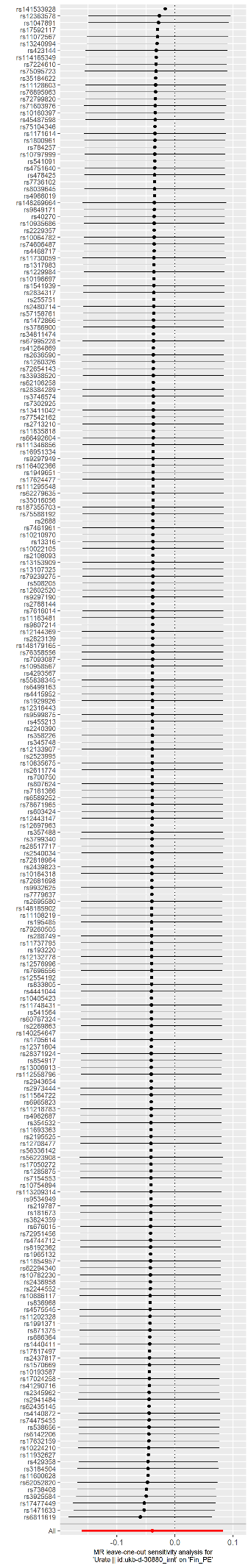

Supplement: Supplementary file 2 — Additional file 2: Supplementary Figure 1. The plots of “leave-one-out” analysis method to show the influence of individual SNP on the causal effect of genetically predicted uric acid (CKDGen consortium) on venous thromboembolism. Supplementary Figure 2. The plots of “leave-one-out” analysis method to show the influence of individual SNP on the causal effect of genetically predicted uric acid (UKB) on venous thromboembolism. Supplementary Figure 3. The plots of “leave-one-out” analysis method to show the influence of individual SNP on the causal effect of genetically predicted uric acid (CKDGen consortium) on deep venous thrombosis. Supplementary Figure 4. The plots of “leave-one-out” analysis method to show the influence of individual SNP on the causal effect of genetically predicted uric acid (UKB) on deep venous thrombosis. Supplementary Figure 5. The plots of “leave-one-out” analysis method to show the influence of individual SNP on the causal effect of genetically predicted uric acid (CKDGen consortium) on pulmonary embolism. Supplementary Figure 6. The plots of “leave-one-out” analysis method to show the influence of individual SNP on the causal effect of genetically predicted uric acid (UKB) on pulmonary embolism. [file 13075_2023_3115_MOESM2_ESM.docx]
